# Correlations between Vascular Stiffness Indicators, OPG, and 25-OH Vitamin D3 Status in Heart Failure Patients

**DOI:** 10.3390/medicina55060309

**Published:** 2019-06-25

**Authors:** Florina Nicoleta Buleu, Constantin Tudor Luca, Anca Tudor, Marius Badalica-Petrescu, Alexandru Caraba, Ana Pah, Doina Georgescu, Ruxandra Christodorescu, Simona Dragan

**Affiliations:** 1 Department of Cardiology, “Victor Babes” University of Medicine and Pharmacy, 300041 Timisoara, Romania; buleu.florina@gmail.com (F.N.B.); marius_badalica@yahoo.com (M.B.-P.); ana11p@yahoo.com (A.P.); simona.dragan@umft.ro (S.D.); 2 Department of Functional Sciences, “Victor Babes” University of Medicine and Pharmacy, 300041 Timisoara, Romania; 3 Department of Internal Medicine, “Victor Babes” University of Medicine and Pharmacy, 300041 Timisoara, Romania; alexcaraba@yahoo.com (A.C.); doinageox@gmail.com (D.G.); ruxandra_christodorescu@yahoo.com (R.C.)

**Keywords:** vitamin D, arterial stiffness, heart failure, renal resistive index, OPG

## Abstract

*Background and objectives*: The purpose of the study is to correlate vascular calcification biomarkers osteoprotegerin (OPG) and 25-hydroxyvitamin D_3_ (25-OH-D_3_), indicators of arterial stiffness carotid-femoral pulse wave velocity (c-f PWV) and renal resistive index (RRI), with parameters of left ventricular function in heart failure patients versus control. *Materials and methods*: Our case-control study compared 60 patients with ischemic heart failure and reduced left ventricular ejection fraction (LVEF) (<40%) with a control group of 60 healthy age-matched subjects (CON). Serum levels of OPG and 25-OH-D_3_ were determined by ELISA. Left ventricular volumes (LVESV, LVEDV) and LVEF were measured by echocardiography. C-f PWV was determined using the arteriograph device. RRI was measured by duplex Doppler. Peak systolic velocity (PSV) and minimum end-diastolic velocity (EDV) were determined using angle correction. The estimated glomerular filtration rate (eGFR) was calculated using the MDRD equation. The Pearson’s correlation coefficient was used for interpretation of results. *Results*: OPG values were significantly higher in heart failure (HF) patients vs. CON (4.7 ± 0.25 vs. 1.3 ± 0.67 ng/mL, *p* < 0.001). 25-OH vitamin D_3_ levels were significantly lower in HF patients vs. CON (20.49 ± 7.31 vs. 37.09 ± 4.59 ng/mL, *p* < 0.001). Multiple regression analysis considering 25-OH D3 as a dependent variable demonstrated indicators of vascular stiffness RRI, c-f PWV and vascular calcification biomarker OPG as predictors. OPG values were significantly correlated with cardiac parameters LVEDV (*r* = 0.862, *p* < 0.001), LVEF (*r* = −0.832, *p* < 0.001), and c-f PWV(*r* = 0.833, *p* < 0.001), and also with 25-OH-D_3_ (*r* = −0.636, *p* < 0.001). RRI values were significantly correlated with cardiac parameters LVEDV (*r* = 0.586, *p* < 0.001) and LVEF (*r* = −0.587, *p* < 0.001), and with eGFR (*r* = −0.488, *p* < 0.001), c-f PWV(*r* = 0.640, *p* < 0.001), and 25-OH-D_3_ (*r* = −0.732, *p* < 0.001). *Conclusions*: This study showed significant correlations between vitamin D deficit and vascular stiffness indicators in heart failure patients with reduced ejection fraction, demonstrating the importance of these examinations for a better evaluation of these patients. Together with the evaluation of renal function, the measurement of vascular stiffness indicators and biomarkers might play a key role in identifying patients at greater risk for worsening disease prognosis and for shorter life expectancy, who could benefit from vitamin D supplementation. The abstract was accepted for presentation at the Congress of the European Society of Cardiology, Munich, 2018.

## 1. Introduction

Arterial stiffness is a term used to define the rigidity of the arterial wall, and also a term that refers to the viscoelastic properties of the vessel wall [1]. Increased central arterial stiffness, a distinctive sign of aging, is associated with cardiovascular disease (CVD), including stroke, coronary heart disease, and heart failure (HF) [2]. In studies, arterial stiffness is closely related to both left ventricular (LV)systolic and diastolic dysfunction. Additionally, people with reduced ejection fraction (HFrEF) have increased arterial stiffness. In HF, a strong predictor of mortality is arterial stiffness [3].

Patients with cardiovascular disease often present low levels of vitamin D. Steroid hormone and vitamin D deficiency has been shown to be associated with chronic HF development in numerous studies [4]. Associated with vascular stiffness [5], vitamin D deficiency is a biomarker of subclinical atherosclerosis, thus being a predictor of cardiovascular morbidity and mortality [6].

Renal insufficiency, common in HF patients, worsens the prognosis [7]. However, morphological changes are often detected late and are unspecific. In recent years, there has been growing data on the clinical relevance of measuring renal resistive index (RRI) for the study of vascular anomalies in the renal parenchyma. Currently, RRI measured in intrarenal segmental arteries is a well-known indicator of renal and interstitial vascular damage that increases cardiovascular risk [8]. RRI values are not specific to a single disease, and in selected groups of patients they seem to be a perfect indicator of cardiovascular changes and also a predictor of rapid loss of renal function. Increased RRI may reflect vascular resistance, depending on the state of the total and local vascular bed. Under specific and well-defined conditions, RRI appears to be a good indicator of vascular damage [8].

Osteoprotegerin (OPG), a glycoprotein involved in phosphocalcic metabolism, considered in studies as an inflammatory biomarker, has been shown to be a biomarker of instability of atherosclerotic plaques, leading to calcification of vessels in patients with cardiovascular disease [9]. Furthermore, increased levels of OPG have been consistently associated with higher incidence and prevalence of coronary artery disease and heart failure [10]. In addition, very low levels of 25-OH-D_3_ have been associated with high levels of OPG and high vascular calcification [11].

A simple, robust, and validated measure of arterial stiffness is represented by pulse wave velocity (PWV) measurement. In short, the shape of arterial blood pressure is a composite of the forward pressure wave created by ventricular contraction and a wave reflected from a distal location. The gold standard measurement of arterial stiffness is carotid-femoral PWV [12,13].

This study aims to correlate 25-OH-D_3_ and vascular calcification biomarker OPG levels with arterial stiffness indicators measured by carotid-femoral PWV and RRI with left ventricular function parameters in heart failure patients versus control.

## 2. Material and Methods

### 2.1. Study Design and Patient Population

This case-control study was conducted between July 2017 and September 2018 in the Cardiovascular Prevention and Rehabilitation Clinic of Institute of Cardiovascular Diseases Timisoara, Romania, on 60 patients with chronic heart failure with reduced LVEF (<40%) consecutive to coronary artery disease (CAD) in stable condition, selected from 482 hospitalized patients with HF of all causes. All heart failure patients included in the study underwent diagnostic coronary angiography, followed by revascularization procedures in selected cases, as documented by individual case histories. Patients with acute heart failure, degenerative valvular diseases, congenital heart diseases, cardiomyopathies of other causes than CAD, pericardial, pulmonary, or rheumatic inflammatory diseases were excluded from the study.

All patients were staged at the time of hospital admission according to the New York Heart Association (NYHA) functional classification, in NYHA class II-IV. The diagnosis was based on typical signs and symptoms, as recommended by European guidelines [14]. The HF patients were compared with a control group of 60 healthy age-matched subjects (CON), selected in the same period from a data base of 3500 subjects screened for cardiovascular risk factors delivered by primary care physicians who collaborate with our clinic. CON was made up of subjects without cardiovascular diseases (CAD, peripheral artery disease, carotid artery disease, HF, or stroke), inflammatory diseases, active infections, or known neoplasms. Also within this group, systemic blood pressure values were controlled without antihypertensive treatment, fasting blood glucose was less than 100 mg/dL, and the lipid profile parameters were within normal limits. The study conformed to the Declaration of Helsinki and was approved by the local ethics committee, and all patients provided written, informed consent. Ethical approval number 1265 (approved on 23 February 2017).

### 2.2. Clinical and Biochemical Evaluation

All patients underwent clinical and biochemical evaluation. Personal data was taken: age, gender, family history of early cardiovascular disease in first-degree blood relatives (for subjects aged 55 years for masculin gender and aged 65 years for feminin gender), and smoking status. The standard objective examination included measuring systolic (SBP), diastolic blood pressure (DBP), and body mass index (BMI). Blood pressure (BP) was determined according to the European Guidelines on cardiovascular disease prevention in clinical practice [15]. A usual tensiometer (Riester, Jungingen, Germany) was used, with a suitable cuff for the arm of each subject. BP was measured on both arms, taking into account the highest value obtained. Body weight (kg) was determined using a mechanical scale. Height (m) was determined using a metal talimeter (Fazzini, Vimodrone, Italy). The BMI was calculated according to the following formula: BMI = weight (kg) ÷ height^2^ (m^2^). For determination of fasting blood glucose, the hexokinase test (HK) was made using Siemens Dimension RXL-MAX, Dade Behring device, Erlangen, Germany and reagents. For determining total cholesterol, triglycerides, LDL and HDL-cholesterol fractions, and photometric methods (Siemens Dimension RXL-MAX, Dade Behring, Erlangen, Germany) were used. For determination of serum creatinine, the Jaffe method without deproteinization was used. Estimated glomerular filtration rate (eGFR) was calculated based on MDRD (Modification of Diet in Renal Disease) formula. eGFR = 186 × (Creatinine/88.4)^−1.154^ × (Age)^−0.203^ × (0.742 if female).

#### 2.2.1. Hydroxyvitamin D_3_ and OPG Analysis

Vitamin D and OPG were determined using commercial ELISA kits. 25-OH-D_3_ was determined using DIAsource 25-OH Vitamin D Total Elisa 90kit (DIAsource ImmunoAssays SA, Louvain-la-Neuve, Belgium),and values were interpreted as follows: a value greater than or equal to 30 ng/mL was considered sufficient, values between 21ng/mL to 29 ng/mL were considered insufficient, the deficit was defined by values between 10 ng/mL and 20 ng/mL, and values less than or equal to 10 ng/mL were defined as severe deficiency. OPG normal range has been established according to the Elabscience Human OPG ELISA Kitused (ORION Biologics Srl, Cluj-Napoca, Romania); levels ≥ 2 ng/mL were considered pathological.

#### 2.2.2. Determination of Echocardiographic Parameters

Transthoracic echocardiography was performed in all patients enrolled in the study on the GE Vivid 9 ultrasound system, manufactured by GEMS Ultrasound, Tirat Carmel, Israel. M-mode, 2D, color flow, and Doppler studies were performed on all patients. M-mode ultrasound displayed the movement of all structures intersecting the beam with time and was used to accurately measure heart structures. Two-dimensional ultrasound provided morphological and functional information about dimensions, cavities, cardiac walls, presence of valvular lesions, ejection fraction, and systolic performance. LV function was assessed qualitatively as normal, or as mild, moderate, or severe dysfunction. Only the patients with reduced LVEF (<40%) were included in the study. The EF by the biplane disc summation method (Simpson’s rule) wascalculated.

#### 2.2.3. Measurement of Arterial Stiffness

Carotid-femoral PWV was performed with the MedexpertArteriograph device version 3.0.0.3 manufactured by TensioMedKft., Budapest, Hungary, which determined both the central pressure, the augmentation index (Aix), and the pulse wave propagation velocity in the aorta (PWVao). The measurements were performed after a minimum period at rest of 10 min in a quiet room in a supine position. The same examiner performed repeated measurements according to the method of determination presented by expert consensus document on arterial stiffness [12]. For a period of at least 3 h prior to the measurements, smoking and foods or drinks with caffeine content were not permitted. At least 10 h prior to the investigation, alcohol consumption was interrupted. Like blood pressure, arterial diameter and arterial stiffness show a circadian variation, increasing during sleep. Therefore, patients were not allowed to fall asleep while determining these parameters. During the measurements, patients did not speak.

#### 2.2.4. Measurement of Renal Vascular Damage

**Figure 1:**
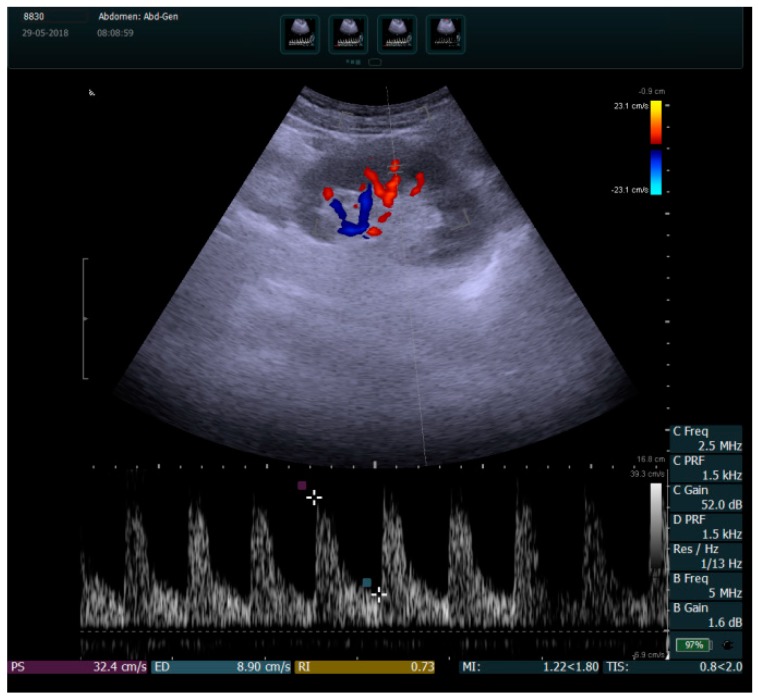
Color duplex sonography of the left kidney intrarenal segmental arteries. In this case, the renal resistive index (RRI) interval is 0.73, measured in a patient with ischemic heart failure.

RRI was measured by duplex Doppler in the intrarenal segmental arteries visualized in color mode (Figure 1). Peak systolic velocity (PSV) and minimum end-diastolic velocity (EDV) were determined using angle correction, and RRI was defined as (PSV-EDV)/PSV. The normal RRI values in adults range between 0.47 and 0.70. A difference of <5–8% can exist between the two kidneys [16]. Measurements were made using the same GE Vivid 9 ultrasound system.

### 2.3. Statistical Analysis

The statistical analysis was made using SPSS v17 software and included descriptive statistics results (mean ± standard deviation, standard error, and confidence intervals). The differences between independent groups were obtained by the Mann–Whitney test, the correlations between numerical variables were made using the multivariate regression model, and the strength of correlation was obtained with the Pearson’s correlation coefficient. Nominal variables were compared and associated by the Pearson’s chi-squared test. Any value of *p* < 0.05 was considered statistically significant.

## 3. Results

Clinical, biochemical, and demographic features of the patients are summarized in Table 1.

No difference between groups was observed regarding age, body mass index, or gender. The mean values of systolic blood pressure, diastolic blood pressure, and heart rate showed no statistically significant difference between the two groups.

There were significant differences between the two groups regarding ejection fraction (27.60 ± 6.26% in HF + CAD vs. 54.50 ± 2.90 in CON, *p* < 0.001), left ventricular end-diastolic volume (178.87 ± 50.63 mL in HF + CAD vs. 82.27 ± 10.50 mL in CON, *p* < 0.001), left ventricular end-systolic volume (109.33 ± 27.78 mL in HF + CAD vs. 37.33 ± 15.39 mL in CON, *p* < 0.001), left ventricular end-diastolic diameter (62.93 ± 10.59 mm in HF + CAD vs. 40.02 ± 7.15 mm in CON, *p* < 0.001), left ventricular end-systolic diameter (47.78 ± 9.96 mm in HF + CAD vs.20.45 ± 2.53 mm in CON, *p* < 0.001), and left atrial volume (46.08 ± 10.58 mL in HF + CAD vs. 23.07 ± 4.88 mL in CON, *p* < 0.001). In the HF + CAD group, T2DM was present in 26 patients (43.33%) and atrial fibrillation in 25 patients (41.66%). No difference between groups was observed regarding TC (*p* = 0.154), LDL-c (*p* = 0.072), and HDL-c (*p* = 0.075). A significant difference between groups was observed in TG levels (178.3 ± 19.24 mg/dL in HF + CAD vs. 112.0 ± 27.8 mg/dL in CON, *p* < 0.001). (Table 1)

**Figure 2:**
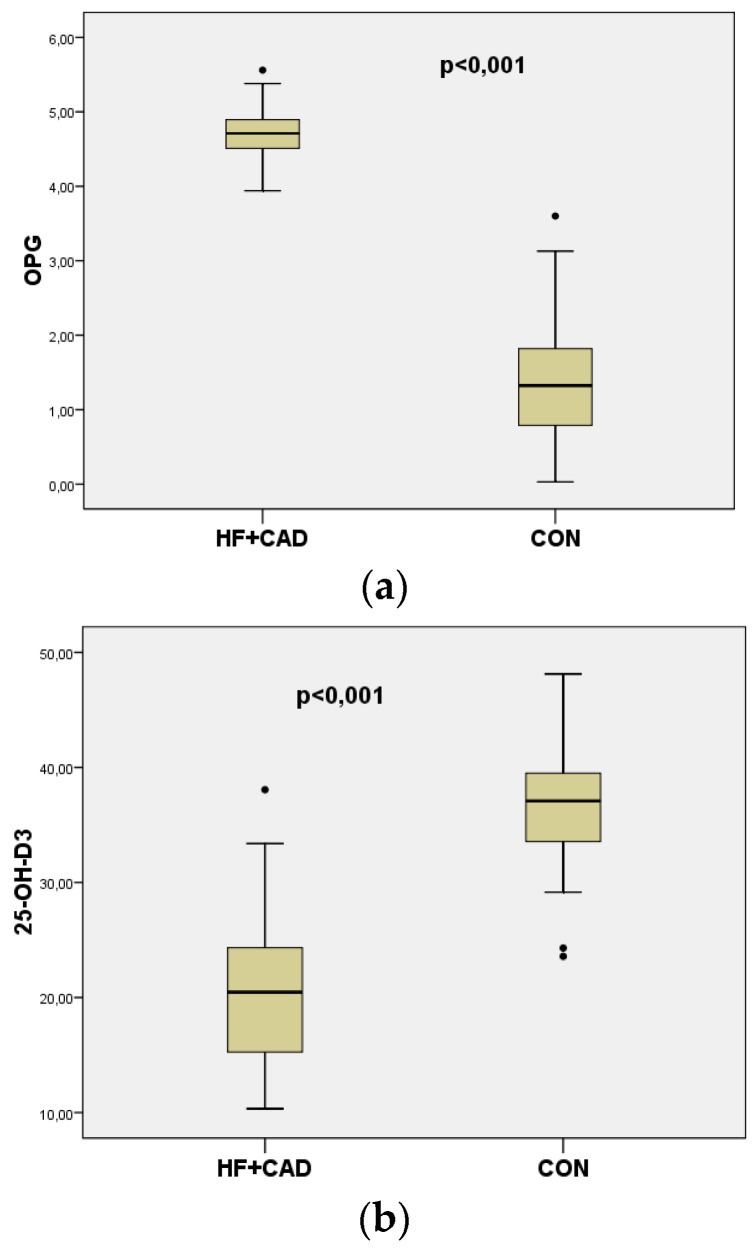
(**a**) Boxplots representing serum levels of OPG in patients with HF+CAD (*n* = 60) and CON (*n* = 60); (*p* < 0.001).(**b**) Boxplots representing serum levels of 25-OH-D_3_ in patients with HF + CAD (*n* = 60) and CON (*n* = 60) (*p* < 0.001). The dots·in Figure 2a,b represent outside values (value is >1.5 times and <3 times the interquartile range beyond either end of the box).

The mean values of OPG were significantly higher in heart failure patients compared to CON (4.7 ± 0.25 vs. 1.3 ± 0.67 ng/mL, *p* < 0.001) (Figure 2a). The mean values of 25-OH-D_3_ were significantly lower in heart failure patients compared to CON (20.49 ± 7.31 vs. 37.09 ± 4.59 ng/mL, *p* < 0.001) (Figure 2b).

**Figure 3:**
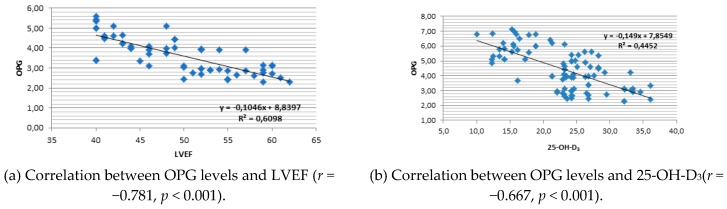

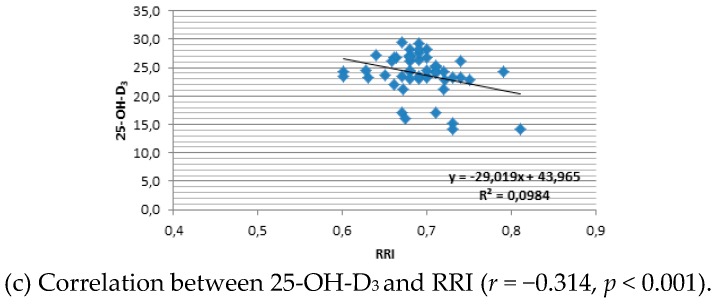
Linear regression plot.

A strong negative correlation was found between OPG values and LVEF (*r* = −0.832, *p* < 0.001) (Figure 3a) and also 25-OH-D_3_ (*r* = −0.636, *p* < 0.001) (Figure 3b). OPG values were positively correlated with c-f PWV (*r* = 0.833, *p* < 0.001) and LVTDV (*r* = 0.862, *p* < 0.001). There was a significant negative correlation between RRI values and 25-OH-D_3_ (*r* = −0.732, *p* < 0.001) (Figure 3c). RRI was also negatively correlated with eGFR (*r* = −0.488, *p* < 0.001).

Multiple regression analysis considering 25-OH D3 as dependent variable demonstrated indicators of vascular stiffness RRI (*p* = 0.004), c-f PWV (*p* = 0.005), and vascular calcification biomarker OPG (*p* = 0.025) as predictors. (Table 2).

Patients with HF+ CAD (*n* = 60) were divided into two subgroups according to vitamin D levels (≥20 ng/mL and <20 ng/mL). Numeric values were compared between the two subgroups using the non-parametric Mann–Whitney test, because values did not respect a normal distribution. Results are presented in Table 3. There was a significant association between vitamin D levels and all variables in the table. Vascular stiffness indicators c-f PWV and RRI were significantly increased in vitamin D deficit (*p* < 0.001). Vascular biomarker OPG was significantly increased according to vitamin D deficit (*p* < 0.001). Cardiac parameters LVEDD, LVESD, LVEDV, and LVESV were also significantly increased in vitamin D deficit (*p* < 0.001, respectively). LVEF was significantly decreased according to vitamin D deficit (*p* < 0.001).

## 4. Discussion

In the present study, we observed that RRI and c-f PWV as indicators of arterial stiffness and serum OPG and 25-OH-D_3_ levels as biomarkers of arterial stiffness were significantly modified in ischemic HF compared to control. Numerous recent studies have demonstrated a relationship between coronary atherosclerosis and OPG levels [9] or between coronary atherosclerosis and 25-OH-D3 levels [17], but no study has associated these biomarkers of arterial stiffness with RRI, an indicator of renal vascular damage common in heart failure patients [16].

In a study conducted on 4063 patients with stable CAD, Bjerre et al. observed that OPG levels were significantly increased in non-survivors (21%). Over the 6-year follow-up period, OPG proved to be a strong predictor of all-cause mortality. They concluded that serum OPG levels had a long-lasting independent predictive power for all-cause mortality and cardiovascular death in these patients [18]. Aksu et al. sustained that increased OPG could be accepted as a biomarker of coronary atherosclerosis [19]. In our study, mean serum OPG levels were higher in patients with HF and CAD compared to CON (*p* < 0.001). These results are in line with similar published findings [18,19,20].

Grandi et al. found that the risk of cardiovascular mortality was increased by 83% in individuals with low 25-OH-D_3_ levels [21]. Al Mheid et al. reported that vitamin D deficit is associated with increased arterial stiffness and endothelial dysfunction in healthy subjects [22].

In our study, the mean serum values of 25-OH-D_3_ were significantly lower in heart failure patients compared to CON (20.49 ± 7.31 vs. 37.09 ± 4.59 ng/mL, *p* < 0.001), as confirmed in previous studies [23,24]. Multiple regression analysis by the Enter method considering 25-OH D3 as dependent variable demonstrated indicators of vascular stiffness RRI (*p* = 0.004) and c-f PWV (*p* = 0.005) as predictors, suggesting that vitamin D deficit might contribute to increased vascular stiffness in heart failure (Table 2). By further refining the comparison within the HF+CAD group (*n* = 60) by applying the non-parametric Mann–Whitney test for numeric variables, we demonstrated that vitamin D deficit is not only significantly associated with increased vascular stiffness but also with significant increased cardiac diameters and volumes, as well as decreased ejection fraction (*p* < 0.001 for all comparisons, Table 3). Arterial stiffness and vitamin D status have been linked in several studies in CAD and in patients with accelerated atherosclerosis due to systemic inflammatory diseases [17,25,26]. However, the relationship between vitamin D and inflammatory markers seems controversial, since high levels of vitamin D are associated with higher levels of C reactive protein in asymptomatic subjects [27].

The role of vitamin D deficit in arterial stiffness is multifactorial, associating inflammation, vascular muscle proliferation, insulin resistance, and regulation of the rennin-angiotensin system, which all contribute to disease progression in HF. A recent meta-analysis of randomized controlled trials (RCTs) on vitamin D supplementation in high-risk cardiovascular patients demonstrated protective effects, improving blood pressure, dyslipidemia, and inflammation [28]. Another meta-analysis that included seven RCTs with a total of 465 patients demonstrated that vitamin D supplementation inhibits ventricular remodeling and improves cardiac function in patients with HF [29]. Additionally, a recent RCT published by Raed et al. demonstrated that arterial stiffness was improved by vitamin D supplementation in a dose-dependent manner, in overweight African Americans [30]. The post-hoc analysis of another very recent study conducted by Zitterman et al. on daily vitamin D supplementation in advanced HF concludes that cardiac function is not significantly improved in all patients, only in those aged >50 years [31].

Cardiac and renal function are closely linked, and communication between these organs occurs through multiple common pathogenetic mechanisms that can cause HF development and aggravation [32]. In terms of cardiovascular risk assessment, the RRI value is an indicator of increased microvascular tonus correlated with the degree of kidney damage of various causes, especially due to high blood pressure. Significant associations between RRI and a variety of cardiovascular risk factors such as left ventricular hypertrophy and carotid atherosclerosis have been described in numerous studies [33,34]. Significant correlations between RRI and aortic stiffness measured by pulse wave velocity have also been reported [35]. These associations demonstrate a close relationship between vascular rigidity in large vessels and in small renal arteries, responsible for increased intrarenal resistance.

The present study showed significantly higher RRI values in HF patients compared to CON (0.71 ± 0.08 vs. 0.61 ± 0.05, *p* < 0.001). We have also observed a significant increase of vascular rigidity expressed by c-f PWV, increased OPG levels as rigidity biomarker, and significant reductions in 25-OH-D_3_ levels in HF patients compared to CON (Table 1). Moreover, the strong negative correlations between LVEF and OPG (*r* = −0.832, *p* < 0.001) are suggestive of the fact that vascular rigidity increases with severity of heart failure. RRI was negatively correlated with eGFR (*r* = −0.488, *p* < 0.001) and with 25-OH-D3 (*r* = −0.732, *p* < 0.001). eGFR is a major determinant of accelerated progression of central and peripheral arterial stiffness. A study conducted by Kwamato et al. on 1716 participants showed that decreased eGFR is associated with an increased PWV in the general population [36]. Moreover, aging also contributes to decrease of eGFR and increased arterial stiffness [12]. We studied a group of HF patients with reduced LVEF vs. controls matched for age and sex, in which RRI, eGFR, and LVEF were significantly decreased and correlated, suggesting that in these patients renal arterial damage and severity of heart failure are strongly interconnected, probably determining each other. The presence of T2DM in 26 patients in the HF group might also have contributed to further explain the increased RRI in these patients, as confirmed by other studies [16].

Our results demonstrate a strong connection between arterial stiffness indicators RRI and PWV with biomarkers OPG and vitamin D in heart failure with reduced ejection fraction. To this day, there are unconclusive data to assess the impact of vitamin D supplementation on indicators of arterial stiffness, due to inappropriate study design, insufficient duration of supplementation, and insufficient power [37,38].

Limitations of the study: This study had a relatively small sample size. We investigated the correlation between vitamin D status and vascular stiffness without focusing on pathophysiological aspects and mechanisms that could explain this association.

## 5. Conclusions

This study demonstrated significant correlations between vitamin D deficit and vascular stiffness indicators in heart failure patients with reduced ejection fraction, demonstrating the importance of these examinations for a better evaluation and follow-up of these patients. Multiple regression analysis considering 25-OH D3 as a dependent variable demonstrated indicators of vascular stiffness RRI, c-f PWV, and biomarker OPG as predictors, suggesting that the correction of vitamin D deficits could improve vascular rigidity. Together with an evaluation of renal function, the measurement of vascular stiffness indicators and biomarkers might play a key role in identifying patients at higher risk for worsening disease prognosis and for shorter life expectancy.

The clinical implications of vitamin D supplementation to improve the life course of these patients are still under scrutiny. Assessment of vitamin D and vascular stiffness in heart failure is needed, because vitamin D deficiency and its consequences for increased cardiovascular risk are well documented. Large, well-designed RCTs are needed to confirm dosage recommendations and benefits, in order to provide a solid base of evidence for guideline recommendations in heart failure.

## Figures and Tables

**Table 1:** Characteristics of all patients (*n* = 120).

Variable	HF + CAD (*n* = 60)	Controls (*n* = 60)	*p*-Value
Age, y	68.43 ± 9.00	66.50 ± 8.92	0.240
Male sex, n (%)	30(50.00)	29(48.33)	0.999
BMI, kg/m2	33.01 ± 3.71	31.63 ± 4.65	0.075
SBP, mm Hg	137.85 ± 28.21	128.03 ± 30.33	0.068
DBP, mm Hg	80.73 ± 13.85	75.98 ± 22.32	0.164
HR, bpm	82.77 ± 22.03	76.55 ± 15.08	0.074
AF, n (%)	25 (41.66)	-	-
T2DM, n (%)	26 (43.33)	-	-
LVEF, %	27.60 ± 6.26	54.50 ± 2.90	<0.001
LVEDV, mL	178.87 ± 50.63	82.27 ± 10.50	<0.001
LVESV,mL	109.33 ± 27.78	37.33 ± 15.39	<0.001
LVEDD, mm	62.93 ± 10.59	40.02 ± 7.15	<0.001
LVESD, mm	47.78 ± 9.96	20.45 ± 2.53	<0.001
IVS, mm	12.34 ± 1.47	9.22 ± 0.85	<0.001
LVPW, mm	12.39 ± 1.59	9.68 ± 0.60	<0.001
LAV, mL	46.08 ± 10.58	23.07 ± 4.88	<0.001
RRI	0.71 ± 0.08	0.61 ± 0.05	<0.001
eGFR, ml/min/1,73m^2^	56.88 ± 25.30	85.31 ± 12.20	<0.001
c-f PWV, m/s	9.75 ± 3.11	7.51 ± 1.28	<0.001
OPG, ng/mL	4.7 ± 0.25	1.3 ± 0.67	<0.001
25-OH-D_3_, ng/mL	20.49 ± 7.31	37.09 ± 4.59	<0.001
TC, mg/dL	182.5 ± 14.64	170.3 ± 13.05	0.154
LDL-c, mg/dL	110.3 ± 6.02	114.6 ± 4.15	0.072
HDL-c, mg/dL	41.9 ± 7.37	44.8 ± 6.45	0.075
TG, mg/dL	178.3 ± 19.24	112.0 ± 27.8	<0.001

BMI, body mass index; SBP, systolic blood pressure; DBP, diastolic blood pressure; HR, heart rate; AF, atrial fibrillation; T2DM, type 2 diabetes mellitus; LVEF, left ventricular ejection fraction; LVEDV, left ventricular end-diastolic volume; LVESV, left ventricular end-systolic volume; LVEDD, left ventricular end-diastolic diameter; LVESD, left ventricular end-systolic diameter; IVS, interventricular septum; LVPW, left ventricular posterior wall; LAV, left atrial volume; RRI, renal resistive index; eGFR, estimated glomerular filtration rate; c-f PWV, carotid-femoral pulse wave velocity; OPG, osteoprotegerin; 25-OH-D_3_, 25-hydroxyvitamin D;. TC, total cholesterol; HDL-c, high-density lipoprotein cholesterol; LDL-c, low-density lipoprotein cholesterol, TG, triglycerides. Values were expressed as mean ± standard deviation (SD).

**Table 2:** Predicted variables of linear regression model (by Enter method) considering 25-OH-D_3_ as dependent variable.

Coefficients ^a^							
Predictors	Unstandardized Coefficients		Standardized Coefficients	t	Sig.	95,0% Confidence Interval for B	
	B	Std. Error	Beta			Lower Bound	Upper Bound
(Constant)	87.882	16.368		5.369	<0.001	55.309	120.455
AGE	0.032	0.049	0.047	0.641	0.524	−0.067	0.130
eGFR	−0.006	0.036	−0.012	−0.167	0.867	−0.077	0.065
LVPW,mm	0.080	0.178	0.032	0.452	0.653	−0.273	0.434
LVEF, %	0.010	0.064	0.018	0.151	0.880	−0.118	0.137
LVEDV,mL	−0.023	0.010	−0.179	−2.253	0.073	−0.044	−0.003
c-f PWVA, m/s	−1.011	0.510	−0.227	−1.983	0.050	−2.025	0.004
OPG,ng/mL	−7.984	0.583	−0.220	−1.687	0.025	−9.150	−6.818
Gender	1.468	0.950	0.117	1.546	0.126	−0.422	3.358
RRI	−71.040	23.802	−0.351	−2.985	0.004	−118.408	−23.673

^a^ Dependent variable: 25-OH-D_3_.

**Table 3:** Comparison of patients with HF+CAD (*n* = 60) according to vitamin D levels (≥20 ng/mL and <20 ng/mL). Non-parametric Mann–Whitney test for numeric variables.

Variable	SampleVit.D	Mean ± Std. Deviation	*p*-Value
LVEDD, mm	≥20	52.86 ± 8.543	<0.001
	<20	63.22 ± 9.669	
LVESD, mm	≥20	39.26 ± 7.741	<0.001
	<20	47.73 ± 9.242	
LVEF, %	≥20	33.48 ± 3.710	<0.001
	<20	24.65 ± 4.740	
LVEDV, mL	≥20	128.77 ± 33.862	<0.001
	<20	191.04 ± 44.566	
LVESV, mL	≥20	73.66 ± 24,361	<0.001
	<20	115.62 ± 22.167	
c-f PWV, m/s	≥20	9.89 ± 1.056	<0.001
	<20	12.01 ± 0.781	
OPG, ng/mL	≥20	3.83 ± 1.069	<0.001
	<20	5.89 ± 0.813	
Creatinine, mg/dl	≥20	1.34 ± 0.428	0.033
	<20	1.45 ± 0.301	
Microalbuminuria, mg/24 h	≥20	43.31 ± 34.941	0.037
	<20	70.81 ± 73.798	
RRI	≥20	0.70 ± 0.020	<0.001
	<20	0.75 ± 0.027	
